# Risk and Protective Factors Associated with Self-Cutting Behavior Among Adolescents at First Contact with the Juvenile Court

**DOI:** 10.1007/s10964-023-01839-x

**Published:** 2023-08-17

**Authors:** Jocelyn I. Meza, Johanna Folk, David Hoskins, Kathleen Kemp, Marina Tolou-Shams

**Affiliations:** 1 Department of Psychiatry and Biobehavioral Sciences, University of California, Los Angeles, USA; 2 Department of Psychiatry and Behavioral Sciences, University of California, San Francisco, USA; 3 Pediatric Psychology Program, UCSF Benioff Children’s Hospital, San Francisco, USA; 4 Department of Psychiatry and Human Behavior, Brown University, Warren Alpert Medical School, Providence, USA; 5 Department of Child and Adolescent Psychiatry and Bradley Hasbro Children’s Research Center, Rhode Island Hospital, Providence, USA

**Keywords:** Self-cutting behavior, Adolescence, Juvenile legal system

## Abstract

Adolescents involved in juvenile legal system are at increased risk for self-cutting behavior, however, correlates associated with elevated risk remain underresearched, particularly among youth with first involvement with the court. This study utilized an epidemiological two-year longitudinal study involving 401 adolescents at first contact with the court (*M*_age_ = 14.47; *SD*_age_ = 1.94 years; 43% female; 42% Latinx/Hispanic) and an involved caregiver. Study aims examined key prospective psychosocial correlates of self-cutting behavior. Baseline assessments captured individual and family level risk and protective factors; self-cutting behavior was assessed longitudinally every four months post-baseline for 24 months. Psychosocial correlates of self-cutting behavior included adolescent affect dysregulation, post-traumatic stress disorder symptoms, impulsive decision making, anxiety and depression symptoms. Significant protective factors included positive communication with caregiver and family, higher self-esteem, and having a caring and supportive family. These findings suggest that internalizing symptoms as well as difficulties with emotion regulation and impulsive decision making are correlated with heightened risk for self-cutting behavior among adolescents involved in the juvenile legal system. The findings also suggest that individual and family level protective factors, like positive communication and a supportive family, are associated with decreased risk for self-cutting behavior among adolescents at first contact with the court.

## Introduction

Non-suicidal self-injury (NSSI) is a public health concern that affects adolescents disproportionately. The typical age of onset for NSSI is during adolescence (between ages 13–16; Muehlenkamp et al., 2018). NSSI includes a wide range of behaviors in which there is no intent to die, with the most common type being self-cutting behavior, and it is estimated that 13–45% of adolescents engage in some form of NSSI (Lloyd-Richardson et al., 2007). Rates of NSSI vary depending on the sample (i.e., community vs. clinical vs. forensic), assessment timeframe (i.e., in the last month vs. previous year vs. lifetime; see Casiano et al., 2013) and based on behaviors included in the NSSI measure (i.e., skin picking). For example, in a large community sample of adolescents, the overall prevalence of NSSI was 31%, however, when the different NSSI behaviors were examined, self-cutting behavior was endorsed by 44% of the sample (Somer et al., 2015). Rates of NSSI are significantly higher among adolescents involved in the juvenile legal system (Casiano et al., 2013; Lüdtke et al., 2018). It is important to note that in large samples of mixed-gender adolescents involved in the juvenile legal system, self-cutting behavior has been reported as the most common type of NSSI behavior (McReynolds et al., 2017). Despite differences in nomenclature, recent data suggests that emergency room visits for NSSI behavior increased for females between the ages of 10–14, with an 18.8% annual increase from 2009 to 2015 (Mercado et al., 2017). NSSI, including self-cutting behavior, is an important mental health issue given that research has consistently shown that it is a robust predictor of future suicide attempts and deaths (Hamza et al., 2015), and this is particularly true among adolescents involved in the juvenile legal system (Koposov et al., 2021). In fact, rates of NSSI among youth in juvenile correctional facilities range between 6.2 to 44% (Casiano et al., 2013). Taken together, the rapid increase in self-cutting behavior prevalence among youth involved in the juvenile legal system and its strong predictive validity with future suicide attempts, self-cutting behavior is a public health concern that warrants more research to help inform preventative interventions (i.e., risk assessment and screening) and diversion from juvenile legal settings. This study addresses this current gap by using a longitudinal sample of court involved adolescents to examine prospective risk and protective factors of self-cutting behavior.

A clear understanding of predictors of self-cutting behavior is an important first step for developing prevention strategies. However, key correlates must be examined within a socioecological framework that critically considers the context in where youth are embedded in. One key setting that has been historically neglected in self-cutting behavior research despite its strong link with severe suicidality outcomes, is the juvenile legal system. The Sequential Interception Model describes the various touchpoints throughout the judicial system that can be used to divert individuals with mental health problems (i.e., self-cutting behavior) into alternative rehabilitative or treatment settings (Munetz & Griffin, 2006). The Sequential Intercept Model has been applied to the juvenile legal system to better understand potential touchpoints of diversion: (1) first contact with emergency services, (2) initial court hearings and detention post-arrest, (3) jails/detention, (4) community re-entry and (5) community corrections (Heilbrun et al., 2017). Understanding the various touchpoints across the juvenile legal system is key in understanding self-cutting behavior and other forms of self-harm given that findings support that risk for suicide increases exponentially with greater involvement with the juvenile legal system. Unfortunately, most research to date that has focused on self-cutting behavior among youth in the juvenile legal system has primarily focused on youth that are already deeply involved in the system (i.e., detention in juvenile facilities and has not focused on diverted youth, which would include youth that have been diverted form incarceration at various touchpoints including contact with law enforcement or court processing (Dauria et al., 2018). Youth that have been diverted from detention/incarceration during their first-time involvement with the juvenile legal system are oftentimes referred to as court-involved, non-incarcerated youth (Tolou-Shams et al., 2019). Examining prevalence rates and correlates of self-cutting behavior among youth that have been diverted from incarceration during their first legal involvement with the court is key for understanding behavioral health needs that need to be addressed as a way of prevention into further legal involvement and worsening self-cutting behavior outcomes.

Adolescents impacted by the juvenile legal system are at particularly high risk for self-cutting behavior due to high rates of mood disorders, substance use, childhood trauma, and impulsivity (see Casiano et al., 2013). The majority of research has focused on suicidality (Hayes, 2009) and less so on the different behaviors that encompass NSSI (see Casiano et al., 2013 and Jin et al., 2021 for exceptions). NSSI correlate studies that focus on high-risk groups, such as adolescents in the juvenile legal system, are urgently needed to inform suicide prevention development. Prior studies involving detained adolescents indicate that psychiatric diagnoses, including a diagnosis of attention-deficit/hyperactivity disorder (ADHD) and post-traumatic stress disorder (PTSD) were linked to both suicidal ideation and attempts, however, this study did not examine correlates associated with risk of self-cutting behavior and included an all-male sample (Ruchkin et al., 2017). Epidemiological studies of adolescents involved with the juvenile court and living in the community, have found that prior offense, substance use, and childhood sexual abuse are the main contributors to elevated risk of suicide ideation and attempts (Kemp et al., 2016). In a study utilizing the present baseline sample, participants that were female, bisexual and endorsed more severe post-traumatic stress symptoms had higher odds of self-cutting behavior (Jin et al., 2021). Similar results have emerged in samples of all female detained adolescents, in where results highlighted that prior traumatic exposure (i.e., childhood maltreatment) was associated with self-cutting behavior (McReynolds & Wasserman, 2011). An authoritative review of suicidal behavior among adolescents in the juvenile legal system concluded that depression, sexual abuse, and trauma are the most commonly identified risk factors. Yet, most studies to date have focused on diagnostic categories (i.e., diagnostic status, like anxiety disorder diagnosis) associated with suicidality (i.e., suicide ideation and attempts) and less attention has been given to the examination of transdiagnostic variables (i.e., affect dysregulation scores) and dimensional scores (i.e., symptoms of PTSD) in longitudinal samples associated with risk of self-cutting behavior. Utilization of transdiagnostic dimensions in longitudinal studies allow for more precise estimates of predictors associated with future elevated risk of self-cutting behavior.

Another significant gap in the literature is that studies to date have also neglected to examine *protective processes* that can decrease the risk of self-cutting behaviors among adolescents impacted by the juvenile legal system. For example, a review highlighted that screening at time of entry into the juvenile legal system was associated with decreased suicide rates (Casiano et al., 2013). Other large clinical samples of adolescents suggest that social support functions as a protective factor for adolescents at risk for NSSI (West, 2019). Large, ethnically-diverse, adolescent samples point to both self-esteem and parental/family support as protective factors for NSSI (Brausch & Gutierrez, 2010). Other studies examining the role of family processes in the risk of NSSI have found that more parental connectedness was present among adolescents who did not self-harm, compared to those adolescents that did engage in self-harm (Taliaferro et al., 2012). However, epidemiological studies with racially and ethnically diverse samples that examine the psychosocial-cultural correlates (both in terms of risk and protection) of self-cutting behavior are scarce. Lack of ethnically diverse samples is a significant gap in current research studies, particularly when Latinx youth are overrepresented in the juvenile legal system. Particularly, it is still unknown whether the correlates related to NSSI among community and clinical samples are the same for adolescents involved in the juvenile legal system. A better understanding of protective processes that can mitigate self-cutting behavior risk among ethnically diverse adolescents at first contact with the court can be used to develop culturally responsive interventions that help divert youth from hospitalization and legal involvement, and can potentially disrupt current inequities (i.e., overrepresentation of incarceration) impacting Latinx youth.

Above and beyond risk and protective factors associated with self-cutting behavior among youth involved in the juvenile legal system, prevalence rates of adolescent self-cutting behavior vary by gender and ethnicity. For example, adolescent girls report significantly higher rates of NSSI compared to adolescent boys (Bresin & Schoenleber, 2015). These gender differences have been replicated across heterogenous samples of adolescents (Guan et al., 2012; Victor et al., 2018), in meta-analyses (Bresin & Schoenleber, 2015) and among adolescents in the juvenile legal system (Lüdtke et al., 2018). Differences across ethnicities for NSSI have been less consistent, particularly when examining Latinx versus non-Latinx samples (Gulbas et al., 2015). Some studies have found that Latinx adolescents are at higher risk and have higher prevalence rates of self-cutting behavior or other forms of NSSI (Chesin et al., 2013; Monto et al., 2018) while other studies report no significant differences between Latinx and non-Latinx groups for NSSI (Guan et al., 2012; see Rojas-Velasquez et al., 2021 for review). These heterogenous findings, along with the scarcity of studies using large ethnically diverse and gender-balanced adolescent samples, warrant further examination of gender and ethnic differences in self-cutting behavior.

## Present Study

Adolescence is a developmental period marked by elevated risk for NSSI, more so among adolescents impacted by the juvenile legal system. Very little research has specifically focused on unfolding the risk and protective factors associated with such elevated risk among justice-impacted adolescents. This study addresses two research questions. First, what are the characteristics of self-cutting behaviors among adolescents with first contact with the juvenile court? More specifically, given mixed findings regarding demographic differences in self-cutting behavior noted above, this study aimed to examine if there were differences in longitudinal rates of self-cutting behaviors (i.e., across all seven timepoints of follow up) among males vs. females and for Latinx vs. non-Latinx adolescents. The hypothesis was that females and non-Latinx adolescents would endorse higher rates of self-cutting behaviors. In addition, given the longitudinal nature of the study, a second study aim was to characterize the different subgroups of self-cutters (i.e., those adolescents that engaged in self-cutting behavior after becoming involved in the juvenile justice system). Lastly, risk and protective factors associated with self-cutting behavior were examined above and beyond sociodemographic characteristics. Given previous findings, the hypothesis was that PTSD and depression symptoms would emerge as key risk factors, while family support and positive self-esteem would emerge as protective factors.

## Methods

### Overview of Procedures

Eligible adolescents: (1) had been in contact with the juvenile court in Rhode Island for the first time within the past 30 days; (2) had a first-time, open status (i.e., offense due to being under <18 years, such as truancy or alcohol use) and/or delinquent (i.e., illicit act regardless of age, such as assault or breaking and entering) petition filed through a large family court in the northeastern U.S. in the last 30 days; (3) had no prior history of juvenile court involvement; and (4) were living in the community. Study exclusion criteria included being younger than 12 or older than 18, having a prior court petition at time of recruitment, cognitive impairment that would impede adolescent or caregiver ability to complete assessments, caregiver’s unwillingness to participate, and/or if the caregiver and adolescent had not lived in the same household for at least the prior six months. Court staff estimates and records indicated approximately 50% of the 4800 adolescents seen at the court setting during the enrollment period (2014–2016) were potentially eligible. The Principal Investigator’s university and collaborating sites’ Institutional Review Boards approved all study procedures. Additional study methods and procedures are described in Tolou-Shams et al., 2019.

Adolescent and caregiver dyads received a study flyer with their court appointment date notification letter and research assistants approached potentially eligible dyads at their first appointment to determine interest and eligibility. Interested adolescent and caregivers were screened for eligibility in a private setting at the court and for those eligible, assent and consent were obtained off-site at the home, private community space, or research lab. Assessments were conducted in private spaces using tablet-based, audio-assisted computerized assessment in English (and in Spanish for caregiver only). Follow-up assessments were conducted every four months post-baseline for two years. The current study uses data from all seven study timepoints. Caregivers and adolescents received $50 for baseline, 12- and 24-month follow-up assessments, with opportunities to earn an additional $20 for a diagnostic interview during year 1 and an additional $30 for 4, 8, 16, 20-month follow-up assessments.

### Participants

Participants included 401 first-time court-involved youth and caregiver dyads who completed baseline and longitudinal follow-up assessments for a 24-month follow-up period in Project EPICC (from June 2014 to April 2016). Of the 401 participants, 56.8% were male and 31.4% identified as non-Latinx White, 10.7% non-Latinx Black, 15% non-Latinx Other/Multi-racial, and 41.9% Hispanic/Latinx (*M*_age_ = 14.47 years, *SD* = 1.94 years). Caregivers of involved adolescent participants were primarily female (87.2%) and 53.0% non-Latinx White and 33.8% Hispanic/Latinx (*M*_age_ = 41.0 years; for more details on the caregiver sample see Folk et al., 2020). About half of the participants (48.4%) were charged with a status offense that would not be considered illegal if an adult committed the same offense (e.g., truancy from school, alcohol use, curfew), and half (51.4%) were charged with a delinquent offense that would be considered illegal regardless of age (e.g., breaking and entering, assault).

### Measures

#### Demographics

Demographics including age, gender, race, and ethnicity were self-reported by adolescents and caregivers.

#### Predictor variables

All predictors were measured during baseline assessment (t1).

### Risk Factors

#### Affective reactivity

Affective reactivity was assessed using six items from the Affective Reactivity Index (ARI; Stringaris et al., 2012). Items are rated by adolescents on a Likert-type scale (0 = not true to 3 = certainly true). A sample item from this measure was “I am easily annoyed by others.” Responses were summed to create a total score with a possible range of 0 to 18; higher scores indicate greater severity of irritability (*α* = 0.897).

#### Impulsive decision making

Impulsive decision making was assessed using the 11 item Impulsive Decision-Making scale (IDM; Donohew et al., 2000). Items are rated by adolescents on a Likert-type scale (1 = never, 2 = sometimes, 3 = often, 4 = always) and summed to yield a total score with a possible range of 11 to 44. A sample item from this measure is “When I do something, I do the first thing that comes to mind.” Higher scores reflect greater impulsivity in decision making (*α* = 0.759).

#### Affect dysregulation

Affect dysregulation was assessed using the six item Affect Dysregulation Scale *(*ADS; Brown et al., 2012). Items refer to the last 4 months and are rated on a frequency Likert-type scale (1 = never to 4 = always). Items included are suggested from the larger SIDES measure (Pelcovitz et al., 1997) but were modified for ease of comprehension for adolescent sample and to reference general feelings, not just anger (Brown et al., 2012). Items were summed to create a total score, which ranged from 6 to 24. A sample item included: “In the past 4 months, small problems got me very upset.” Higher scores indicate greater affect/emotions dysregulation. (*α* = 0.882).

#### Trauma exposure and posttraumatic stress symptoms

Trauma exposure and posttraumatic stress symptoms were assessed using adolescent ratings on the 9-item National Stressful Events Survey PTSD Short Scale (NSESSS; LeBeau et al., 2014). Adolescent reported experiences of posttraumatic stress symptoms on a Likert-type scale (1 = not at all to 5 = extremely). An additional response option (6 = “I have never experienced a stressful event”) was used to identify adolescent with no trauma exposure. For adolescents who answered any item with “I have never experienced a stressful event,” the entire scale was recoded as missing. Prorated scores were calculated when no more than two items were left unanswered (sum of items answered x total number of items on measure)/number of items answered, rounded to the nearest whole number). A sample item included: “Feeling very emotionally upset when something reminded you of a stressful experience.” The total symptom score (possible ranges 9 to 45) was used, with higher scores indicating greater PTSD symptom severity (*α* = 0.946).

#### Internalizing symptoms

Internalizing symptoms were assessed via adolescent and caregiver ratings on the 148-item Behavior Assessment System for Children-2 (BASC-2; Kamphaus & Reynolds, 2007). Parents and adolescents were asked to rate symptoms based on the last two weeks. Items are rated on a Likert-type scale (1 = never to 4 = almost always). Scores are summed to create a raw score, which is then converted to a t-score (standardized scores with *M* = 50 and *SD* = 10) based on a general adolescent sample. T-scores of 59 and below are considered “within normal limits”, 60 to 69 are “at-risk”, and 70 and above are in the clinically significant range. The current study used the adolescent and caregiver t-scores for Anxiety and Depression subscales, which were analyzed separately.

#### Alcohol and other drug use

Alcohol and other drug use was assessed by asking adolescents if they used alcohol or other drugs (e.g., cocaine) in the last 4 months (yes/no) on the Adolescent Risk Behavior Assessment (ARBA; Donenberg et al., 2001).

#### Anxiety or depression diagnosis

Diagnosis of anxiety or depression were also self-rated by asking adolescents if they had a diagnosis of anxiety or depression in the last 4 months (yes/no) on the Adolescent Risk Behavior Assessment (ARBA; Donenberg et al., 2001).

### Protective Factors

#### Positive aspects of communication

Adolescents reported positive aspects of communication with their caregiver on the Parent-Adolescent General Communication Scale (PAC; Barnes & Olson, 1985). The positive aspects of communication subscale includes seven items that are rated on a Likert-type scale (1 = strongly disagree to 5 = strongly agree). A sample item is: “My parent tries to understand my point of view.” The seven items are summed to create a total subscale score, with higher scores indicating more positive communication. Possible scores in this subscale ranged from 7 to 35 (*α* = 0.911).

#### Self-concept and family support resilience factors

Self-concept and family support resilience factors were assessed via the 62-item Youth Resiliency: Assessing Developmental Strengths Scale (YRADS; Donnon & Hammond, 2007). The 62 items are used to measure the 10 factors, or 31 development strengths subscales associated with the resiliency framework. The current study used the following subscales: *self-efficacy* (believing in one’s abilities to do many different things well; 2 items, *α* = 0.70), *self-esteem* (feeling positive about oneself and the future; 2 items, *α* = 0.72), *caring family* (2 items, *α* = 0.86), *family communication* (2 items, *α* = 0.76) and *family support* (2 items, *α* = 0.81). Items are rated on a Likert-type scale (1 = strongly agree to 5 = strongly disagree). The scale was reverse recoded to match the original measure and scores were translated into percentages (5 = 100%, 4 = 75%, 3 = 50%, 2 = 25%, and 1 = 0%). All subscale scores are averages ranging from 0 to 100 and can be interpreted as: 0–24 = not aware of the strength and not using it (Significant Challenge), 25–49 = becoming aware of the strength but is not using it in their lives (Moderate Challenge), 50–74 = understands the strength and is starting to use it in their lives (Moderate Strength) and 75–100 = understands and actively uses the strength (Significant Strength). Self-efficacy and self-esteem are two developmental strengths listed in the self-concept resiliency factor. Caring family (defined as the family providing a nurturing, caring, loving home environment), family support (defined as the family providing trust, support, and encouragement regularly), and family communication (defined as the adolescent being able to communicate with family openly about issues/concerns) are three developmental strengths from the family support resiliency factor.

#### Criterion variables

Measures of self-cutting behavior were assessed at baseline (t1) and every 4 months for 24 months (t2–t7).

#### Self-cutting behavior

Self-cutting behavior was assessed using the Functional Assessment of Self-Mutilation (FASM; Lloyd et al., 1997). Adolescent self-reported endorsement of self-cutting behavior using a single item: “In the last 4 months have you intentionally cut your body using pins, knives, razorblades, safety pins, or other things?” with yes/no as response options. For analyses we created a dichotomous variable reflecting positive endorsement of self-cutting behavior during any of the follow-up points (yes = 1, no = 0).

#### Patterns of self-cutting behavior

To assess repetition of self-cutting behaviors across the 4-month assessments, not engaging in self-cutting behavior at either assessment time-point was dummy-coded as 0 and endorsement of self-cutting behavior as 1. Based on self-cutting behavior endorsements across baseline and the follow-up assessments, adolescents were classified as: controls (never endorsed self-cutting behavior at either baseline or any of the follow-up assessments); self-cutting behavior initiators (denied self-cutting behavior at baseline but endorsed self-cutting behavior between t2–t7); self-cutting behavior desisters (endorsed self-cutting behavior at t1 but denied self-cutting behavior at all other t2–t7), and self-cutting behavior repeaters (endorsed self-cutting behavior both at baseline/t1 and at least once between t2–t7).

##### Covariates

To ascertain whether the correlates of interest are related specifically to self-cutting behaviors rather than to confounding factors, we statistically adjusted for baseline measures that have been empirically associated with the predictors of interest and the criterion measures of self-cutting behavior, including (a) adolescent’s ethnicity (Latinx/Hispanic vs. non-Latinx/Hispanic), (b) gender, and (c) age.

### Data Analytic Plan

Statistical analyses were performed with SPSS for Mac, Version 24. First, bivariate correlations among all study variables of interest were conducted. Next, a series of chi-squared tests were used to assess differences between Latinx and non-Latinx adolescents with respect to both baseline (t1; assesses any previous lifetime self-cutting behavior) and longitudinal (endorsed self-cutting behavior between t2 to t7) self-cutting behavior. Next, chi-square tests were conducted to assess differences between males and females with respect to self-cutting behavior outcomes. Effect sizes were calculated using odds ratios (ORs). The criterion variables included two self-cutting behavior time points: previous lifetime self-cutting behavior endorsed at baseline (t1) and longitudinal self-cutting behavior, which included any endorsement of self-cutting behavior between timepoints t2 to t7. In order to provide a more comprehensive picture, descriptive analyses examined the prevalence rates of self-cutting behaviors across the seven time points; and also report on the self-cutting behavior classifications (i.e., non-self-cutting group [never endorsed self-cutting behavior at any timepoint], self-cutting behavior initiators [denied self-cutting behavior at baseline/t1 but endorsed self-cutting behavior between t2 to t7], self-cutting behavior desisters [endorsed self-cutting behavior at t1/baseline but denied self-cutting behavior at all other timepoints assessed between t2 and t7], self-cutting behavior repeaters [endorsed self-cutting behavior at both baseline/t1 and on at least one other assessment time point between t2 and t7]).

Second, key risk factors of self-cutting behavior were examined using a series of binary logistic regressions to test whether baseline/t1 affective reactivity, impulsive decision making, affect regulation, PTSD symptoms, adolescent rated anxiety, adolescent rated depression, caregiver rated anxiety, caregiver rated depression, depressive disorder diagnoses in the last 4 months, anxiety disorder diagnoses in the last 4 months, and drug use in the last 4 months, independently predicted self-cutting behavior (endorsed between t2 to t7) over and above sociodemographic variables (Step 1: covariates; Step 2: twelve predictors of interest entered individually). To examine key protective factors, binary logistic regressions were conducted to test whether baseline/t1 adolescent rated positive aspects of communication with caregiver, positive self-esteem, self-efficacy, perceived family support, family communication and perception of having a caring family, independently predicted self-cutting behavior (endorsed between t2 to t7) over and above sociodemographic variables (Step 1: covariates; Step 2: six predictors of interest entered individually). Adjustment for multiple comparisons was conducted by controlling for false discovery rate using the Benjamini–Hochberg (BH) procedure (Benjamini & Hochberg, 1995). The false discovery rate (FDR) was set at *p* < 0.10 because a liberal criterion is recommended when the cost of a false negative is high (i.e., not detecting a significant predictor of self-harm; see McDonald, 2014). Other studies examining self-harm outcomes have also set an FDR *p* < 0.10 (see, for example, Hooijer & Sizoo, 2020). A series of *t*-tests for continuous variables and chi-square tests for dichotomous variables were conducted between those with positive self-cutting behavior histories and those without across our eighteen predictors of interest. Effect sizes were computed using Cohen’s *d*.

Very little data was missing for predictors, covariates, and the outcome variables of interest (i.e., 1–4%), so none were imputed. The only exception was adolescent rated data for positive aspects of communication (7.5%, *n* = 30 missing), and these missing cases were excluded from the analyses. However, these participants’ data did not differ on any variables of interest (*p*s < 0.05).

## Results

### Intercorrelations and Descriptive Analyses

Table 1 presents the intercorrelations among study variables. As expected, longitudinal ratings of self-cutting behavior were significantly and moderately associated with all predictors of interest (*ps* < 0.01), with the exception of impulsive decision making (*p* = 0.344). All predictors of interest were in the expected direction, such that risk factors were *positively* associated with longitudinal ratings of self-cutting behavior (correlation ranges between *r* = 0.16 to 0.39), while proposed protective factors were negatively associated with longitudinal ratings of self-cutting behavior (correlations ranges between *r* = −0.14 to −0.25).

**Table 1:** Intercorrelations among all study variables

Study Variables	1	2	3	4	5	6	7	8	9	10	11	12	13	14	15	16	17
1. Self-cutting behavior	–																
2. Caregiver BASC Anxiety T-Score	0.10	–															
3. Caregiver BASC Depression T-Score	0.13^**^	0.73^**^	–														
4. Adolescent BASC Anxiety T-Score	0.31^**^	0.36^**^	0.35^**^	–													
5. Adolescent BASC Depression T-Score	0.32^**^	0.27^**^	0.41^**^	0.75^**^	–												
6. Depressive Disorder Diagnosis	0.20^**^	0.21^**^	0.24^**^	0.23^**^	0.29^**^	–											
7. Anxiety Disorder Diagnosis	0.25^**^	0.23^**^	0.22^**^	0.29^**^	0.31^**^	0.85^**^	–										
8. Affective Reactivity Index	0.18^**^	0.24^**^	0.33^**^	0.54^**^	0.57^**^	0.06	0.10^*^	–									
9. Impulsive Decision Making	0.09	0.06	0.13^*^	0.22^**^	0.29^**^	0.07	0.05	0.39^**^	–								
10. SIDES-Affect Regulation Scale	0.26^**^	0.22^**^	0.32^**^	0.61^**^	0.57^**^	0.24^**^	0.19^**^	0.58^**^	0.31^**^	–							
11. NSESSS Total Score	0.25^**^	0.26^**^	0.30^**^	0.67^**^	0.60^**^	0.22^**^	0.21^**^	0.53^**^	0.27^**^	0.63^**^	–						
12. Drug/Alcohol Use (4 Months)	0.16^**^	0.02	0.09	0.09	0.11^**^	0.04	0.05	0.03	0.05	0.07	0.06	–					
13. Positive Aspects of Communication	−0.23^**^	0.20	−0.05	−0.14^**^	−0.29^**^	−0.11	−0.10	−0.21^**^	−0.24^**^	−0.22^**^	−0.10	−0.06	–				
14. YRADS Self-Esteem	−0.25^**^	−0.10	−0.22^* *^	−0.40^**^	−0.52^**^	−0.17^**^	−0.19^**^	−0.36^**^	−0.21^**^	−0.45^**^	−0.38^**^	−0.06	0.34^**^	–			
15. YRADS Self-Efficacy	−0.14^**^	−0.13^*^	−0.18^* *^	−0.28^**^	−0.36^**^	−0.11^*^	−0.08	−0.22^**^	−0.15^**^	−0.30^**^	−0.26^**^	−0.04	0.24^**^	0.63^**^	–		
16. YRADS Family Support	−0.19^**^	0.02	−0.13 ^*^	−0.27^**^	−0.39^**^	−0.15^**^	−0.15^**^	−0.37^**^	−0.28^**^	−0.41^**^	−0.26^**^	−0.10	0.55^**^	0.66^**^	0.51^**^	–	
17. YRADS Family Communication	−0.19^**^	−0.02	−0.14^* *^	−0.27^**^	−0.37^**^	−0.12^*^	−0.11^*^	−0.34^**^	−0.29^**^	−0.36^**^	−0.21^**^	−0.08	0.53^**^	0.60^**^	0.52^**^	0.83^**^	–
18. YRADS Caring Family	−0.18^**^	0.02	−0.14 ^*^	−0.23^**^	−0.35^**^	−0.08	−0.09	−0.38^**^	−0.28^**^	−0.37^**^	−0.22^**^	−0.06	0.52^**^	0.57^**^	0.47^**^	0.85^**^	0.82^**^

*Notes*. *BASC* Behavior Assessment System for Children -2; *IR* Interpersonal Relations; *SIDES* Structured Interview for Disorders of Extreme Stress; *NSESSS* National Stressful Events Survey *PTSD* Short Scale; *YRADS* Youth Resiliency: Assessing Developmental Strengths

^*^p < 0.05, ^**^p < 0.01

Bivariate analyses (Table 2a) revealed that 21.4% (*n* = 85) of the overall sample endorsed lifetime self-cutting behavior at baseline/t1, with significant differences between males (10.2%, *n* = 23) and females (36.3%, *n* = 62; *χ*^2^[1, N = 397] = 39.35, *p* < 0.001, OR: 5.02, 95% CI: 2.95, 8.55). In addition, significant differences emerged for longitudinal ratings of self-cutting behavior for the overall sample (17.8% endorsed between t2 to t7; *n* = 64) between males (7.5%, *n* = 15) vs. females (30.8%, *n* = 48); *χ*^2^[1, 356] = 32.58, *p* < 0.001, OR: 5.48, 95% CI: 2.93, 10.26). Bivariate analyses between Latinx and non-Latinx adolescents (Table 2b) revealed marginally significant differences between Latinx (17.9%, *n* = 30) vs. non-Latinx adolescents (25.6%, *n* = 57) for lifetime self-cutting behavior at baseline/t1; *χ*^2^[1, 391] = 3.29, *p* < 0.070, OR: 0.63, 95% CI: 0.39, 1.04. A similar pattern emerged for the longitudinal ratings of self-cutting behavior outcomes, such that Latinx adolescents endorsed significantly lower self-cutting behavior ratings (11.7%, *n* = 18) vs. non-Latinx adolescents (22.6%, *n* = 45); *χ*^2^[1, 352] = 7.18, *p* = 0.007, OR: 0.45, 95% CI: 0.25, 0.82).

**Table 2:** Criterion variable characteristics of overall sample

a Criterion variable characteristics of overall sample, with contrasts between males and females					
Variable	Overall Sample *N* = 397	Male *n* = 226	Female *n* = 171		
	% (*n*)	% (*n*)	% (*n*)	*p*^a^	OR [95% CI]
Self-Cutting Behavior (Baseline)	21.4% (*n* = 85)	10.2% (*n* = 23)	36.3% (*n* = 62)	0.000	5.02 [2.95, 8.55]
	Overall Sample *N* = 356	Male *n* = 200	Female *n* = 156		
Self-Cutting Behavior (Longitudinal)	17.6% (*n* = 63)	7.5% (*n* = 15)	30.8% (*n* = 48)	0.000	5.48 [2.93, 10.26]

b Criterion variable characteristics of overall sample, with contrasts between latinx vs. non-latinx sample					
Variable	Overall Sample *N* = 391	Latinx *n* = 168	Non-Latinx *n* = 224		
	% (*n*)	% (*n*)	% (*n*)	*p*^b^	OR [95% CI]
Self-Cutting Behavior (Baseline)	22.3% (*n* = 87)	17.9% (*n* = 30)	25.4% (*n* = 57)	0.070	0.63 [0.39, 1.04]
	Overall Sample *N* = 352	Latinx *n* = 154	Non-Latinx *n* = 198		
Self-Cutting Behavior (Longitudinal)	17.8% (*n* = 63)	11.7% (*n* = 18)	22.6% (*n* = 45)	0.007	0.45 [0.25, 0.82]

Missing data notes: three participants did not report on gender identity and one participant was missing baseline self-cutting behavior data; and nine participants did not report on Latinx status, and one participant was missing baseline self-cutting behavior data

*OR* odds ratio

^a^Male vs. Female. Significance: Chi-square statistic

^b^Latinx vs. Non-Latinx. Significance: Chi-square statistic

The following self-cutting behavior prevalence rates (i.e., self-cutting behavior in the last four months) were found across the longitudinal assessment time points (see Table 3): 9.0% (*n* = 36 at t1), 9.6% (*n* = 30 at t2), 6.9% (*n* = 21 at t3), 7.4% (*n* = 23 at t4), 7.1% (*n* = 21 at t5), 6.5% (*n* = 18 at t6) and 7.4% (*n* = 22 at t7). In terms of the self-cutting behavior longitudinal classifications, descriptive analyses indicated most adolescents (*n* = 122) never endorsed self-cutting behavior, and the second largest group endorsed prior self-cutting behavior in their lifetime but did not report self-cutting behavior at baseline (*n* = 96). A small proportion denied self-cutting behavior at baseline (t1) but began self-cutting behavior engagement at t2 (“self-cutting behavior initiators”; *n* = 19), whereas *n* = 23 adolescents endorsed self-cutting behavior at t1 but denied self-cutting behavior throughout the t2 to t7 assessments (“self-cutting behavior desisters”), and *n* = 24 adolescents endorsed “repetitive self-cutting behavior” by endorsing self-cutting behavior at both t1 and in at least one other time point between t2 and t7. Longitudinal classifications do not add up to the sample of 401 given missing data at follow-up assessments.

**Table 3:** Criterion variable characteristics across assessment timepoints

	T1	T2	T3	T4	T5	T6	T7
Self-Cutting Behavior Prevalence % (*N*)	9.0% (*n* = 36)	9.6% (*n* = 30)	6.9% (*n* = 21)	7.4% (*n* = 23)	7.1% (*n* = 21)	6.5% (*n* = 18)	7.4% (*n* = 22)
Completed/Valid *N*	400	311	304	311	294	278	299
Missing *N*	1	90	97	90	107	123	102

*Note*. *T* timepoint

### Baseline Predictors of Longitudinal Self-Cutting Behaviors: Binary Logistic Regressions and Cohen’s d

Binary logistic regressions were used to assess longitudinal predictors of self-cutting behavior (endorsed between t2 to t7). For risk factors of self-cutting behavior, after covarying adolescent’s ethnicity (Latinx vs. non-Latinx), gender, and age at baseline, and after adjusting for multiple comparisons, only affect regulation (*p* < 0.001; *d* = 0.69), PTSD symptoms (*p* < 0.001; *d* = 0.63), adolescent rated BASC Anxiety T-scores (*p* < 0.001; *d* = 0.84), and adolescent rated BASC Depression T-scores (*p* < 0.001; *d* = 0.87), affective reactivity (*p* = 0.023; *d* = 0.46), impulsive decision making (*p* = 0.012; *d* = 0.240) positively predicted self-cutting behavior (see Table 4). Self-rated depression (*p* = 0.010; OR = 4.56) and anxiety (*p* = 0.003; OR = 7.54) diagnoses in the last 4-months were also associated with higher risk for self-cutting behavior. For protective factors, after covarying adolescent’s ethnicity (Latinx vs. non-Latinx), gender, and age at baseline, and after adjusting for multiple comparisons, only adolescent ratings of positive aspects of communication with their caregiver (*p* < 0.001; *d* = 0.60), self-esteem (*p* = 0.005; *d* = 0.67), adolescent rated perceptions of family support (*p* = 0.005; *d* = 0.49), adolescent rated perceptions of family communication (*p* = 0.005; *d* = 0.50), and adolescent ratings of having a caring family (*p* = 0.004; *d* = 0.48) were associated with fewer endorsements of longitudinal history of self-cutting behavior (see Table 4).

**Table 4:** Baseline Correlates of Self-Cutting Behaviors between T2 to T7

	No Self-Cutting Behavior *N* = 296 M (SD)/%	Any Self-Cutting Behavior *N* = 64 M (SD)/%	*p*^*a*^	Cohen’s *d*/OR [95% CI]	*p* with covariates^b^
Risk Factors					
Affective Reactivity Index	4.11 (3.57)	5.77 (3.62)	*p* = 0.001	0.46	*p* = 0.023^*^
Impulsive Decision Making	25.06 (5.02)	26.22 (4.57)	*p* = 0.091	0.24	*p* = 0.012^*^
SIDES-Affect Regulation Scale	12.27 (4.11)	15.15 (4.57)	*p* = 0.000	0.69	*p* = 0.001^*^
NSESSS Total Score	9.14 (8.94)	15.11 (10.68)	*p* = 0.000	0.63	*p* = 0.000^*^
BASC Anxiety T-Score (Adolescent Rated)	48.72 (11.99)	59.25 (114.56)	*p* = 0.000	0.84	*p* = 0.000^*^
BASC Depression T-Score (Adolescent Rated)	50.74 (12.72)	62.33 (16.03)	*p* = 0.000	0.87	*p* = 0.000^*^
BASC Anxiety T-Score (Caregiver Rated)	51.07 (12.96)	54.42 (14.01)	*p* = 0.065	0.26	*p* = 0.233
BASC Depression T-Score (Caregiver Rated)	57.79 (13.89)	62.44 (15.22)	*p* = 0.018	0.33	*p* = 0.180
Depressive Disorder Diagnosis in Last 4 months	4.4%	17.5%	*p* = 0.000	4.56 [1.94, 10.72]	*p* = 0.010^*^
Anxiety Disorder Diagnosis in Last 4 months	2.7%	17.5%	*p* = 0.000	7.54 [2.89, 19.63]	*p* = 0.003^*^
Psychiatric Hospitalization in Last 4 months	4.8%	13%	*p* = 0.019	2.88 [1.15, 7.19]	*p* = 0.092
Alcohol/Drug Use in Last 4 months	3.4%	12.5%	*p* = 0.003	4.04 [1.53, 10.70]	*p* = 0.049
Protective Factors					
Positive Aspects of Communication	26.11 (6.62)	22.10 (6.72)	*p* = 0.000	0.60	*p* = 0.000^*^
YRADS Self-Esteem	74.30 (22.71)	58.47 (27.33)	*p* = 0.000	0.67	*p* = 0.005^*^
YRADS Self-Efficacy	71.96 (21.95)	63.69 (22.97)	*p* = 0.008	0.37	*p* = 0.042
YRADS Family Support	72.90 (22.56)	61.68 (23.92)	*p* = 0.001	0.49	*p* = 0.005^*^
YRADS Family Communication	69.86 (23.32)	58.20 (23.81)	*p* = 0.000	0.50	*p* = 0.005^*^
YRADS Caring Family	72.72 (23.50)	61.31 (24.25)	*p* = 0.001	0.48	*p* = 0.004^*^

*Notes*. *SIDES* Structured Interview for Disorders of Extreme Stress; *NSESSS* National Stressful Events Survey *PTSD* Short Scale; *BASC* Behavioral Assessment System for Children; *YRADS* Youth Resiliency: Assessing Developmental Strengths

^a^Independent samples *t*-test for continuous variables and chi-square statistic for dichotomous variables

^b^Binary logistic regression; Covariates included: adolescent’s ethnicity (Latinx/Hispanic vs. non-Latinx/non-Hispanic), gender and age at baseline

^*^Significant after correction for false discovery rate (Benjamini & Hochberg, 1995)

## Discussion

Self-cutting behavior, a precursor to other self-injurious behaviors including suicide attempts, disproportionately impacts adolescents involved in the juvenile legal system. Despite elevated risk for self-cutting behavior, scarce research has been conducted to examine transdiagnostic and prospective correlates across domains (i.e., individual and family level). More concerning is the historical underrepresentation of samples that include adolescents across the various touchpoints in the juvenile legal system, with most studies to date focusing on self-cutting behavior among detained/incarcerated youth. To address this gap, this study investigated an epidemiological and longitudinal sample of never incarcerated adolescents involved in the juvenile court for the first time with two major goals: (a) characterizing longitudinal patterns of self-cutting behavior and (b) ascertaining risk and protective factors across domains associated with self-cutting behavior. First, overall prevalence rates of previous lifetime histories of self-cutting behavior (assessed at baseline [t1]) were 21.4% and 17.6% for self-cutting behavior rates assessed longitudinally between the seven time points. This is the first study to report on longitudinal rates of self-cutting behavior among non-incarcerated adolescents at their first court involvement. In general, self-cutting behavior rates are consistent with previous large studies of detained adolescents, that report a 25.7% lifetime prevalence of NSSI, with the self-cutting behavior being the most frequent type (13.7%) endorsed by the sample (McReynolds et al., 2017). Still other juvenile legal samples of detained youth have reported rates of NSSI between 10 to 40% (Casiano et al., 2013). Examination of self-cutting behavior among adolescents *across* the various touchpoints of the juvenile legal system, not just during detention (i.e., in a locked facility), can inform key diversion strategies to help adolescents avoid formal penetration into the juvenile legal system and into treatment that addresses their mental health need.

A key component of equitable suicide prevention for youth involves contextualizing surveillance data by examining self-cutting behavior prevalence rates by different sociodemographic factors, like race/ethnicity and gender (Meza et al., 2022). Results regarding gender differences were consistent with previous studies that have also found that adolescent girls engage in higher rates of self-cutting behavior when compared to adolescent boys (Bresin & Schoenleber, 2015; Casiano et al., 2016). However, examination of gender differences in self-cutting behavior among justice involved samples is scarce. Findings from this study that 36.3% of court involved girls and 10.2% of boys engaged in prior self-cutting behavior at baseline are consistent with results from a recent study of court involved adolescents living in the community that found that 43% of girls and 14% of boys endorsed lifetime NSSI (Conrad et al., 2022). These findings are also in line with previous results from the present sample that found that first-time court involved girls were more than three times more likely to report self-cutting behavior histories prior to court involvement (Jin et al., 2021). Study findings also uncovered significant differences between the Latinx and non-Latinx group, such that the Latinx group endorsed significantly *lower* rates of self-cutting behavior when compared to the non-Latinx groups. These results support the study hypothesis that the Latinx group would report lower rates of self-cutting behavior. A previous study of a representative sample of U.S. adolescents found similar rates of NSSI for both Hispanic/Latinx and White adolescents, with reported rates of 19.19 and 17.71%, respectively (Monto et al., 2018). It should be noted that categorization of Latinx vs. non-Latinx status in this study might have obscured some of the differences that have been reported in other studies because we grouped all Latinx adolescents together regardless of their race (i.e., Black Latinx, which historically have lower rates of NSSI when compared to White adolescents; Monto et al., 2018).

Regarding risk correlates examined in this study, findings suggest that the most robust baseline predictors of longitudinal self-cutting behavior were affect dysregulation, PTSD symptoms, impulsive decision making, affective reactivity, and adolescent ratings of internalizing symptoms (anxiety and depression). Study findings are largely consistent with previous studies utilizing adolescent/young adult samples (Adrian et al., 2019; Kranzler et al., 2016) and among samples of justice-involved adolescents (Conrad et al., 2022; Koposov et al., 2021). These findings are not surprising given that theories have postulated that adolescents engage in self-cutting behavior as a way to regulate and cope with distressing emotions (Laye-Gindhu & Schonert-Reichl, 2005). A large body of research examining risk factors for self-cutting behavior have largely focused on emotion dysregulation, with few studies focusing on affective reactivity, which has been implicated as a key mechanism explaining the link between psychopathology and NSSI in adolescent samples (Nock et al., 2008). Findings from this study that both emotion dysregulation and affective reactivity are predictive of self-cutting behavior are therefore well aligned with previous findings. To contribute to the literature, we examined the predictive association between self-rated impulsive decision making with self-cutting behavior, given that previous studies have primarily focused on behavioral measures of impulsivity and have not examined more discrete components of impulsivity, like decision making. Study findings that self-rated impulsive decision making predicts self-cutting behavior extends previous findings that impulsive decision making (measured via task performance) during exposure to critical comments is associated with more frequent NSSI among adults (Allen et al., 2019). Similarly, it was unsurprising that impulsive decision making predicted self-cutting behavior, given that measures of impulsivity have been consistently reported as a putative risk factor for self-cutting behavior in adolescent samples (Cassels et al., 2020; Lockwood et al., 2017). Findings from this study also extend previous results that report cross-sectional associations between baseline PTSD symptoms and self-cutting behaviors, and highlight that baseline PTSD symptoms predict the longitudinal course of self-cutting behaviors (Jin et al., 2021). Taken together, these findings indicate that PTSD symptoms persist in predicting self-cutting behavior, over and above key sociodemographic variables, like age and ethnicity. One key limitation from previous studies is the use of diagnostic categories (i.e., anxiety disorder diagnostic status) and use of cross-sectional samples, which preclude the examination of prospective associations. Results from this study contribute to the literature by offering more precise predictions of self-cutting behavior outcomes, levering prospective analysis of dimensional and transdiagnostic predictors.

Development of effective treatments for self-cutting behavior requires both the reduction of risk factors and the promotion of key protective factors. Analyses from this study focused on the examination of theory driven and evidence-based risk and protective factors. When examining protective factors among our first-time court legally-involved adolescent sample, this study found that adolescent perceptions of a positive home environment characterized by perceived family support and positive communication were very important. For example, findings indicate that positive aspects of communication with parents/caregivers as well as with family members and viewing family as caring and supportive were associated with significantly lower rates of self-cutting behavior. This finding is consistent with previous studies that have examined family environments and have found that perceived parental criticism and low support are predictive of NSSI (Baetens et al., 2015). Given the significant importance of family environment on NSSI, interventions that found promising effects for reducing NSSI included parent and family components, including teaching parents how to validate their adolescents’ emotions (Glenn et al., 2015). Findings examining positive self-esteem as a protective factor also contribute to the current knowledge base, given the historically mixed findings between high self-esteem and negative outcomes (i.e., delinquency) for adolescents involved in the legal system (Kaplan, 1975a). In general, study findings that high self-esteem is associated with reduced risk for self-cutting behavior support previous findings utilizing adolescent samples (Cawood & Huprich, 2011; Garisch & Wilson, 2015). In fact, a review of treatments for self-cutting behavior among adolescents found that the treatment intervention with the most support was the Cutting Down Program, which had significant effects in reducing frequency of NSSI, as well as reduction in depressive symptoms and improvement in self-esteem (Calvo et al., 2022). Taken together, these findings support the target of self-esteem in treatment interventions for adolescent self-cutting behavior. However, targeting improvement in self-esteem in interventions with adolescents involved in the legal system warrant further examination, given that some studies have reported that high self-esteem may be linked to reoffending among adolescent girls (Thapa et al., 2021).

These findings should be interpreted in light of a few limitations. First, this study sample included adolescents that were court involved but had not been detained; as such, no conclusions can be drawn with regard to predictors of self-cutting behavior among detained adolescents. Future research is urgently needed to examine predictors of self-cutting behavior and other forms of self-harm among adolescents across the Sequential Intercept Model (i.e., detention, community re-entry) and whether diversion or recidivism plays a role in their self-cutting behavior. Second, this study assessed self-cutting behavior using a single item that only assessed one type of NSSI. Frequency and severity are two important dimensions of self-harm that warrant further examination, particularly among justice involved samples. The measure of self-cutting behavior likely provided an underestimate of self-harm among this sample. Third, given the small subsample of adolescents indicating self-cutting behavior between the different assessment timepoints, the examination of predictors of the repeated self-cutting behavior was not conducted—an area in need of investigation. Fourth, given data limitations due to small sample sizes across ethnic/racial subcategories (i.e., Black and Latinx participants), post-hoc or sensitivity analyses were not conducted. Finally, risk factors were considered independently in the regression analyses due to the moderate to high correlations between some of the variables of interest (and risk of multicollinearity), and although correction for multiple tests was used, the possibility that Type I errors occurred cannot be ruled out. The effect sizes (e.g., odds ratios and Cohen’s d) of study findings, however, were emphasized as opposed to statistical significance alone. Fifth, methodological constraints have been documented when examining the Latinx population as a homogenous group, such as the lack of generation categorizations (e.g., foreign born vs. U.S. born) and racial identifiers (e.g., White vs Black; Tapia, 2015). Further exploration is needed to solidify whether the heterogeneity and intersectionality of the Latinx sample (e.g., Black Latinx and sexual gender minority status) prove to have distinct self-cutting behavior trajectories. Finally, assessment of gender identity beyond just male/female binary were limited. Adolescents involved in the court who identify as gender-diverse need further attention, particularly given previous findings highlighting their increased risk for self-cutting behavior (Jin et al., 2021).

## Conclusion

Scarcity of research examining transdiagnostic and prospective associations with self-cutting behavior among adolescents with their first-time involvement in the juvenile court prevents the timely and urgently needed development of prevention strategies (i.e., screening or risk assessment tools) that can divert adolescents away from the legal system and into appropriate access of mental health care. To address this gap, it is important to first understand prospective risk and protective factors associated with self-cutting behavior, especially because at-risk adolescents typically do not seek professional help when engaging in self-cutting behaviors. Similar rates of self-cutting behavior among early justice involved and detained adolescents further highlight the importance of early targeted prevention efforts for self-cutting behavior at the point of first contact with the juvenile legal system. Utilizing a transdiagnostic approach, screening tools can be enhanced to improve risk stratification allowing multiple systems with limited resources (e.g., schools, family, courts) to target interventions to adolescents most at risk and further improve access to evidence-based care for a vulnerable and underserved population. Beyond addressing risk factors, prevention efforts will better serve adolescents by looking at the whole person through enhancing protective factors, including the integration of family communication and support.
